# Comparison between community-acquired pneumonia and post-obstructive pneumonia associated with endobronchial tumors

**DOI:** 10.1186/s12890-024-03409-8

**Published:** 2024-11-28

**Authors:** Wenwen Yu, Yubo Shi, Qingsong Zheng, Jianwu Chen, Xie Zhang, Ali Chen, Zhiyang Yu, Weilong Zhou, Li Lin, Legui Zheng, Hua Ye, Yunlei Li

**Affiliations:** 1 Department of Respiratory and Critical Care Medicine, Affiliated Yueqing Hospital of Wenzhou Medical University, Wenzhou, 325600 Zhejiang China; 2 Department of Oncology, Wenzhou Central Hospital of Wenzhou Medical University, Wenzhou, 325000 Zhejiang China; 3 Department of Pathology, Affiliated Yueqing Hospital of Wenzhou Medical University, Wenzhou, 325600 Zhejiang China

**Keywords:** Post-obstructive pneumonia, Community acquired pneumonia, Endobronchial tumor

## Abstract

**Background:**

Endobronchial tumors can infiltrate the bronchial wall or protrude into the bronchial lumen, causing post-obstructive pneumonia (POP). Differentiating between POP and community-acquired pneumonia (CAP) is challenging due to similar clinical, laboratory, and imaging findings, which can delay the diagnosis and treatment of endobronchial tumors.

**Methods:**

We compared general demographic information, laboratory test results, lung CT images, bronchoscopic observations, pathological findings between the POP group and the CAP group.

**Results:**

(1) The POP group consisted mainly of older individuals (mean age 69 vs. 56 years; *P* < 0.05), males (93.4% vs. 47.1%; *P* < 0.05), and smokers (67.2% vs. 14.7%; *P* < 0.05). Clinical symptoms varied, with chest pain (23.0% vs. 11.8%; *P* < 0.05) and hemoptysis (26.2% vs. 10.8%; *P* < 0.05) more prevalent in the POP group. MSCT showed that bronchial wall thickening, bronchial stenosis, occlusion, obstructive emphysema, mucoid impaction, and endobronchial shadows occurred more frequently in POP, while consolidation and exudation shadows were predominant in CAP (*P* < 0.05). (2) In the POP group, neoplasms were the most frequent bronchoscopic findings (57 cases, 93.44%), especially in the upper lungs. Squamous cell carcinoma was the primary pathological type (52 cases, 85.25%). The average delay in diagnosing endobronchial tumors was 214.8 days. In the POP group, 34 cases (55.74%) had abnormal CT images in the past and did not undergo bronchoscopy, resulting in delayed diagnosis. (3) Factors such as gender, age, bronchial occlusion, stenosis, mucus embolism, and intraluminal shadow were determined to be independent risk factors for endobronchial tumors (*P* < 0.05 and OR > 1).

**Conclusions:**

Endobronchial tumors combined with POP are easily misdiagnosed as CAP in the early stage. Factors like bronchial occlusion, stenosis, mucus embolism, and intraluminal shadows on MSCT are significant independent risk factors for these tumors, indicating the need for early bronchoscopy.

## Introduction

Post-obstructive pneumonia (POP) is a type of pulmonary infection that arises due to bronchial obstruction, which may be caused by lung and tracheobronchial tumors, intratracheal foreign bodies, among other factors [1]. In advanced lung cancer patients, POP is common, with incidence rates ranging from 40 to 55%, leading to a poor prognosis and elevated mortality [2]. Especially in central type lung cancer, early-stage intraluminal obstruction can often be treated effectively if detected early. However, the subtle onset and nonspecific symptoms of early-stage lung cancer make it difficult to differentiate from community-acquired pneumonia. Notably, malignancies are identified in about 2% of patients admitted with community-acquired pneumonia [3], posing a significant risk of misdiagnosis or delayed diagnosis [4]. Thus, this retrospective analysis is designed to discern the prevalent clinical, imaging, and bronchoscopic features of POP associated with endobronchial tumors versus CAP, aiming to identify endobronchial tumors as early as possible through characteristic CT imaging and enhance the early detection of endobronchial tumors and improve outcomes.

## Methods

### Study design and participants

This retrospective, single-center cohort study was conducted at Affiliated Yueqing Hospital of Wenzhou Medical University, Zhejiang Province, China. A total of 163 adult patients (aged ≥ 18 years) were enrolled from April 2014 to December 2022. All participants were diagnosed with pneumonia based on Multi-slice Spiral computed tomography (MSCT) findings and received antibiotic treatment. The patients were divided into two groups according to the bronchoscopic biopsy results: 61 with post-obstructive pneumonia and 102 with community-acquired pneumonia. Inclusion criteria included: (1) patients showing inflammatory changes on CT scans with comprehensive clinical and imaging data, and (2) those who underwent bronchoscopy. Exclusion criteria encompassed: (1) coagulation dysfunction or severe hematologic conditions, (2) malignant arrhythmias, (3) organ dysfunction or failure (heart, brain, kidney, lung, or other systemic organs), (4) severe pulmonary hypertension, (5) mental health disorders, and (6) individuals with bronchiectasis, chronic obstructive pulmonary disease, or a history of lung surgery, (7) foreign body in the bronchus. Community-acquired pneumonia definition that these are the diagnostic criteria: “Community-acquired pneumonia was defined by the presence of new or worsening pulmonary infiltrates, along with at least two of the following symptoms: subjective or documented fever (> 37.4°C), increased cough, sputum production, dyspnea, pleuritic chest pain, confusion, crackles, leukocytosis (WBC count > 12,000 cells/µL), or leukopenia (< 6,000 cells/µL)”. Post-obstructive pneumonia was identified by radiographic evidence of a pulmonary infiltrate beyond an obstructed bronchus.

### Data collection

Clinical and demographic data were collected and are detailed in Table 1. This data included age, sex, height, weight, smoking status, medical history, and symptoms such as cough, expectoration, hemoptysis, chest pain, dyspnea, wheezing, and fever. At admission, a comprehensive set of tests was documented: complete blood count, procalcitonin (PCT), C-reactive protein (CRP), arterial blood gas analysis, and tumor markers including carcinoembryonic antigen (CEA), cytokeratin 19 fragment (CYFRA21-1), squamous cell carcinoma antigen (SCC), and neuron-specific enolase (NSE), were assessed to investigate potential oncological causes of post-obstructive pneumonia. Lung imaging was performed using UCT 710 60-slice and Philips Ingenuity 64-slice CT scanners, capturing the entire lung from apex to base. Bronchoscopic evaluations were conducted using an Olympus BF-Q290 bronchoscope to inspect the trachea, bronchi, and distal bronchi, with biopsies taken of any detected abnormalities for subsequent histopathological analysis.

**Table 1:** Comparison of patient data between the POP group and the CAP group

Characteristic	POP group (*n* = 61)	CAP group (*n* = 102)	*P* Value
Demographics and comorbidities			
Age, y^a^	69(58–80)	59(38–80)	**< 0.001**
Body mass index, kg/m2^a^	21.58 ± 2.45	22.11 ± 3.27	0.282
Male sex	57(93.4)	48(47.1)	**< 0.001**
Smoking	41(67.2)	15(14.7)	**< 0.001**
Comorbidities^*^	30(49.2)	60(58.8)	0.257
Clinical features			
Cough	52(85.2)	94(92.2)	0.190
Sputum	42(68.9)	85(83.3)	**0.034**
Hemoptysis	16(26.2)	11(10.8)	**0.016**
Chest pain	14(23.0)	12(11.8)	**0.049**
Dyspnea	9(14.8)	15(14.7)	1.000
Wheezing	5(8.2)	9(8.8)	1.000
Temperature > 37.4 °C	9(14.8)	47(46.1)	**< 0.001**
Laboratory features			
C-reactive protein, mg/L^a^	8.35(5.00-102.86)	36.96(5.54–94.63)	0.228
Procalcitonin, ng/mL^a^	0.25(0.09–0.25)	0.25(0.05–0.25)	**0.013**
White blood cell count, cells/µL^a^	7.93(5.62–9.03)	7(5.42–9.79)	0.325
Lymphocyte count, cells/µL^a^	1.34(1.11–1.94)	1.4(1.15–1.78)	0.715
Neutrophil Count, cells/µL^a^	5.65(3.72–6.79)	4.54(3.09–7.22)	0.469
Platelet count, cells/µL^a^	228.5(196.50-315.75)	263(196–319)	0.907
Globular value, g/L^a^	128.5(117.5-139.25)	126(112–135)	0.075
CEA, ng/mL^a^	2.73(2.03–4.62)	1.67(1.23–2.42)	**< 0.001**
SCC, ng/mL^a^	1.59(1.91)	1.04(0.78–1.41)	**< 0.001**
CYFRA21-1, ng/mL^a^	4.06(2.56–6.04)	1.98(1.55–2.89)	**< 0.001**
NSE, ng/mL^a^	12.90(10.21–17.27)	13(10.91–15.19)	0.853
Radiographic features			
Consolidation	7(11.5)	46(45.1)	**< 0.001**
Exudation	30(49.2)	90(88.2)	**< 0.001**
Emphysema	8(13.11)	1(1.0)	**0.002**
Bronchial wall thickening	12(19.7)	2(2.0)	**< 0.001**
Bronchial occlusion	40(65.6)	8(7.8)	**< 0.001**
Bronchial-stenosis	32(52.5)	16(15.7)	**< 0.001**
Atelectasis	7(11.5)	7(6.9)	0.388
Bronchial mucus embolism	19(31.1)	3(2.9)	**< 0.001**
Shadow in the lumen of the bronchi	29(47.5)	8(7.8)	**< 0.001**
Pleural effusion	2(3.3)	10(9.8)	0.213
Lymphadenectasis	15(24.6)	34(33.3)	0.291

Data are shown as No. (%) of patients and refer to values at the time of admission, unless stated otherwise

Abbreviations: CEA, carcinoembryonic antigen; SCC, squamous cell carcinoma antigen; CYFRA21-1, cytokeratin 19 fragment; NSE, neuron-specific enolase

^*^Comorbidities include hypertension, diabetes, nephritis, coronary atherosclerosis, extrathoracic malignancies, and rheumatic autoimmune diseases

^a^ Median (interquartile range)

### Statistical analysis

Statistical analysis was conducted using IBM SPSS Statistics Software (version 27.0; IBM, New York, USA). Data were tested for normality and variance homogeneity. Normally distributed continuous variables were reported as mean ± standard deviation and analyzed using Student’s t-test. Skewed data were presented as medians (interquartile ranges) and assessed using the Mann-Whitney U test. Categorical variables were evaluated using the χ² test or Fisher’s exact test between the POP and CAP groups. Logistic regression models were used to identify risk factors for endobronchial lesions, excluding variables without significant intergroup differences. *P* < 0.05 was deemed statistically significant.

## Results

### Comparison of clinical and demographic data between patients with post-obstructive pneumonia and community-acquired pneumonia

Clinical and demographic data were collected and are summarized in Table 1. Post-obstructive pneumonia with endobronchial tumors was more common in elderly individuals (69 years vs. 56 years; *P* < 0.05), males (93.4% vs. 47.1%; *P* < 0.05), and smokers (67.2% vs. 14.7%; *P* < 0.05). Both groups displayed symptoms of cough and expectoration with no significant difference (*P* < 0.05). However, hemoptysis and chest pain were more frequent in the post-obstructive pneumonia group (26.2% vs. 10.8% and 23.0% vs. 11.8%, respectively; *P* < 0.05). In contrast, sputum production and fever were more prevalent in the community-acquired pneumonia group (68.9% vs. 83.3% and 14.8% vs. 46.1%, respectively; *P* < 0.05). The post-obstructive pneumonia group showed significantly higher levels of carcinoembryonic antigen, squamous cell carcinoma antigen, and cytokeratin 19 fragment compared to the community-acquired pneumonia group. There was no significant difference in procalcitonin, C-reactive protein, and neuron-specific enolase levels between the groups. More instances of bronchial wall thickening (19.7% vs. 2%; *P* < 0.05) and stenosis (52.4% vs. 15.6%; *P* < 0.05) were observed in the post-obstructive group. In contrast, bronchial occlusion (65.5% vs. 7.8%; *P* < 0.05), intraluminal masses (47.5% vs. 7.8%; *P* < 0.05), and bronchial mucus embolism (31.1% vs. 2.9%; *P* < 0.05) were more prevalent in the community-acquired pneumonia group, which also showed a higher incidence of pulmonary consolidation and/or exudative changes. Pleural effusion and lymphadenopathy were present in both groups without a significant difference.

### Subgroup analysis of patients with post-obstructive pneumonia

**Fig. 1:**
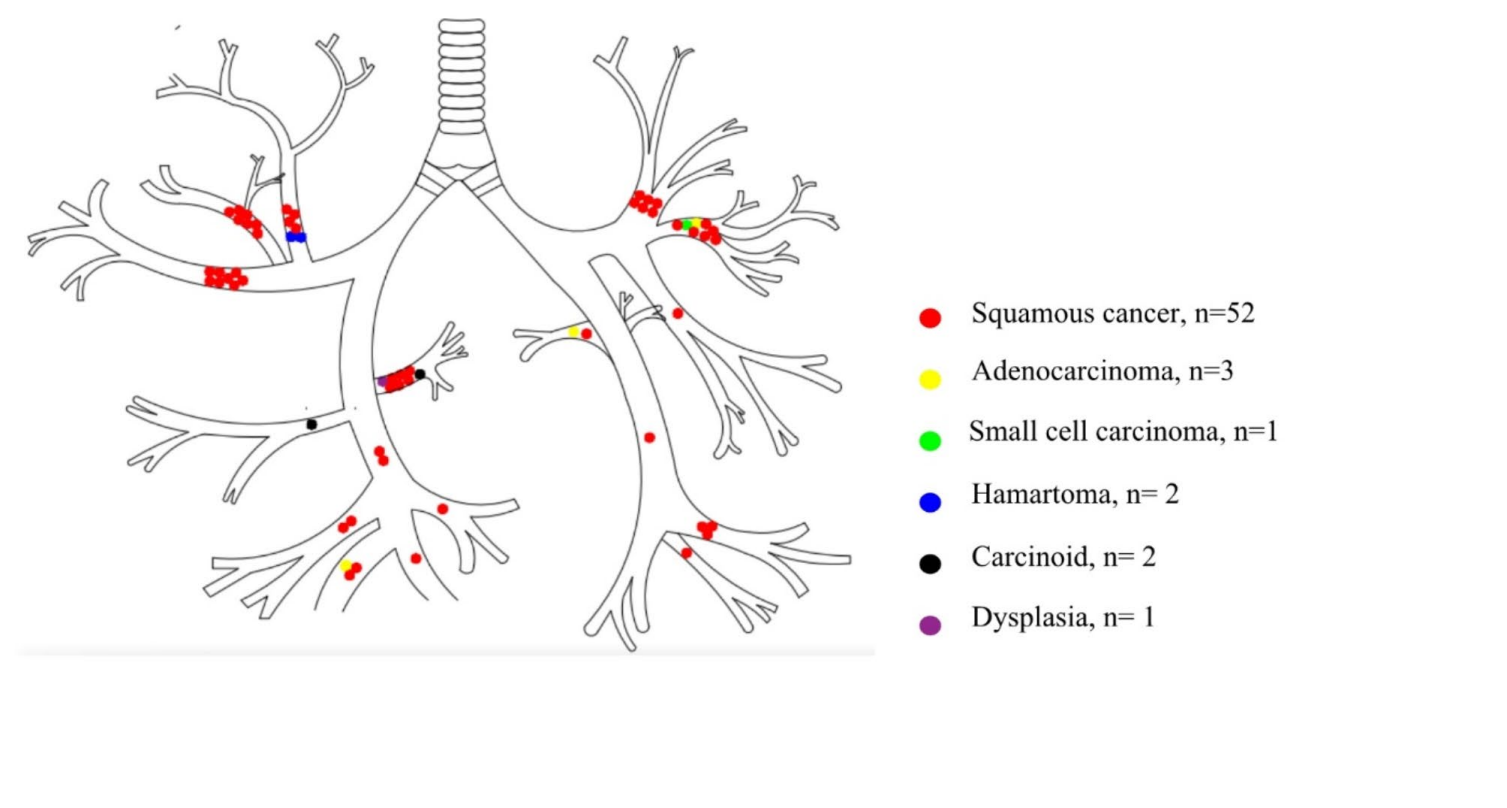
Pathological types and anatomical locations of endobronchial tumors in patients with POP

Bronchoscopy confirmed the presence of malignant tumors, hamartomas, or dysplasia in patients with post-obstructive pneumonia. Among the patients with post-obstructive pneumonia, 39 (63.93%) cases involved the right lung, with 21 cases (34.43%) specifically affecting the right upper lobe. The left lung was involved in 22 cases (36.07%), with the left upper lobe involved in 15 cases (24.59%) (Table 2; Fig. 1).

**Table 2:** Bronchoscopic findings in the POP group

Site		POP group (*n* = 61) (n, %)
Right upper lobe	Anterior segment	8, 13.11%
	Apical segment	6, 9.84%
	Posterior segment	7, 11.48%
	Right middle lobar bronchus	1, 1.64%
Right inferior lobe	Dorsal segment	8, 13.11%
	Basal segment	9, 14.75%
Left upper lobe	Anterior segment	8, 13.11%
	Intrinsic bronchus	6, 9.83%
Left upper lobe	Lingular bronchi	1, 1.64%
Left inferior lobe	Basal segment	5, 8.20%
	Dorsal segment	2, 3.28%

**Fig. 2:**
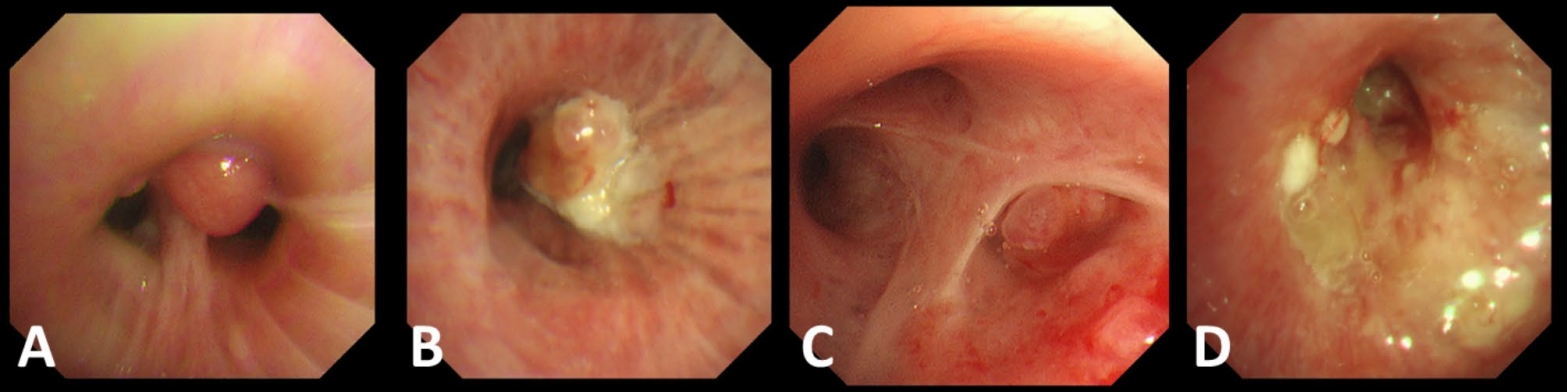
(**A**) Bronchoscopy demonstrated a round, smooth-surfaced mass obstructing the bronchial opening of the upper lobe apical segment of the right lung. (**B**) Bronchoscopy demonstrated a cauliflower-like neoplasm at the opening of the right lower lobe bronchus. (**C**) Bronchoscopy demonstrated a smooth-surfaced neoplasm in the bronchial lumen of the right upper posterior segment, completely obstructing the airway. (**D**) Bronchoscopy demonstrated a rough, congested bronchial wall in the posterior segment of the right upper lobe, accompanied by luminal narrowing

Bronchoscopy identified bronchial infiltration, stenosis, occlusion, and intraluminal neoplasms in the POP group, noting bronchial occlusion in 56 cases (91.8%), intraluminal neoplasms in 57 cases (93.44%), bronchial infiltration in 24 cases (39.34) and bronchial stenosis in 5 cases (8.20%). Pathology revealed squamous cell carcinoma in 52 cases (85.25%), adenocarcinoma in 3 (4.92%), hamartoma in 2 (3.28%), carcinoid in 2 (3.28%), small cell lung cancer in 1 (1.64%), and dysplasia in 1 (1.64%) (Fig. 1). In Fig. 2, we present images of endobronchial tumors from four cases in the POP group. Figure A demonstrated a round, smooth-surfaced mass obstructing the bronchial opening of the upper lobe apical segment of the right lung (Fig. 2A). The mass was biopsied and pathologically confirmed as hamartoma. Figure B demonstrated a cauliflower-like neoplasm at the opening of the right lower lobe bronchus (Fig. 2B), while Figure C demonstrated a smooth-surfaced neoplasm in the bronchial lumen of the right upper posterior segment, completely obstructing the airway (Fig. 2C). Figure D demonstrated demonstrated a rough, congested bronchial wall in the posterior segment of the right upper lobe, accompanied by luminal narrowing (Fig. 2D). The tumors in Figures B-D were all diagnosed as squamous cancer.

Univariate logistic regression analysis was applied to statistically significant variables, with those showing *P* < 0.05 and OR > 1 progressing to multivariate analysis. This analysis identified elderly individuals, males, and CT imaging findings of bronchial obstruction, bronchial stenosis, bronchial mucus embolism, and intrabronchial shadows as independent risk factors for endobronchial tumors (*P* < 0.05), as shown in Table 3.

**Table 3:** Risk factors of endobronchial tumor complicated with POP

	Index	Single factor OR	*P* Value	Multiple factor OR	*P* Value
Demographics	Gender	16.03	**<** 0.001	12.335	**0.014**
	Age	1.088	**<** 0.001	1.289	**0.011**
	Smoking history	1.078	**<** 0.001	1.034	0.072
Laboratory features	PCT	2.386	0.132		
	CEA	1.925	**<** 0.001	1.195	0.495
	SCC	1.425	0.011	1.034	0.904
	CYFRA21-1	1.426	**<** 0.001	1.248	0.181
Radiographic features	Consolidation	0.158	**<** 0.001		
	Exudation	0.129	**<** 0.001		
	Emphysema	15.245	0.011	14.253	0.065
	Bronchial wall thickening	12.245	0.001	5.283	0.167
	Bronchial occlusion	22.381	**<** 0.001	61.349	**0.001**
	Bronchial stenosis	5.931	**<** 0.001	11.032	**0.001**
	Bronchial mucus embolism	14.929	**<** 0.001	20.858	**0.013**
	Shadow in the lumen of the bronchi	10.648	**<** 0.001	5.758	**0.038**

The time from the first appearance of imaging abnormalities in CT to the diagnosis of endobronchial tumors through bronchoscopy is defined as the delay time. If an abnormality is reported by CT and immediate hospitalization or outpatient bronchoscopy is performed, the delay time is 0. We found that the average delay time in the POP group is 214.8 days (range: 0-1170 days). In the POP group, 34 patients (55.74%) had abnormal CT images in the past and did not undergo bronchoscopy, resulting in delayed diagnosis. Thus, prompt recognition of specified imaging characteristics is vital to reduce diagnostic delays. In the post-obstructive pneumonia group, bronchial occlusion imaging showed high sensitivity (65.5%) and specificity (92.2%). Sensitivity increased to 96.7% when combined with bronchial stenosis, but no significant enhancement was noted with other imaging features(Table 4). In the POP group, 24 patients underwent surgical resection, while 2 patient with bronchial hamartoma and 1 patient with bronchial dysplasia received bronchoscopic high-frequency electric loop resection. The remaining patients did not undergo surgery due to factors such as tumor staging or financial constraints.

**Table 4:** Diagnostic value of single and combined imaging findings in endobronchial tumor

Radiographic features	Sensitivity (%)	Specificity(%)	Jorden index
Bronchial occlusion	65.5	92.2	0.577
Bronchial-stenosis	52.5	84.3	0.368
Bronchial mucus embolism	31.1	97.1	0.282
Shadow in the lumen of the bronchi	47.5	92.2	0.397
Occlusion/Stenosis	96.7	78.4	0.751
Occlusion//Shadow in the lumen	73.8	79.4	0.532
Occlusion/Shadow in the lumen	82.0	88.2	0.702
Sstenosis/Occlusion/Shadow in the lumen	98.4	77.5	0.759
Stenosis/Occlusion/Bronchial mucus embolism	98.4	78.4	0.768
Stenosis/Occlusion/Shadow in the lumen/Bronchial mucus embolism	98.4	77.5	0.759

## Discussion

According to the WHO International Agency for Research on Cancer, lung cancer is the leading cause of cancer-related morbidity and mortality worldwide [5]. This is largely due to 85% of patients being diagnosed at advanced stages, missing early diagnostic and treatment opportunities [6]. Lung cancer primarily affects older men, with smoking as the foremost risk factor, although the influence of other factors like environmental pollution and exposure to radioactive substances is on the rise [7].

Endobronchial lesions are potential precursors to central airway lung carcinomas. Early identification and treatment of these lesions can prevent their progression to invasive carcinoma. Of the 61 patients with obstructive pneumonia and endobronchial tumors, 57 were male, and 41 had a smoking history. Univariate analysis identified gender and smoking as independent risk factors for endobronchial tumors, aligning with prior research. While quitting smoking reduces lung cancer risk, former smokers still face a risk nine times greater than non-smokers [8]. Chiaki Endo et al. [9] analyzed 251 patients with early-stage central airway lung cancer, 207 of whom underwent surgical resection. They reported 5-year and 10-year survival rates post-treatment of 96.7% and 94.9%, respectively. Early diagnosis and thorough resection of central pneumonia are crucial for enhancing lung cancer survival rates [10].

MSCT is essential in assessing endobronchial lesions, providing valuable information on tracheal stenosis, bronchial wall thickening, obstruction, tracheobronchial malacia, and both benign and malignant bronchial tumors. Its application in screening high-risk individuals significantly lowers the relative risk of dying from lung cancer [11]. Guidelines recommend MSCT for early diagnosis and screening in high-risk groups [12]. Nonetheless, MSCT may overlook endobronchial lesions, highlighting the need for integrating characteristic imaging features with bronchoscopy to ensure early lesion detection.

Tumor growth within the bronchial lumen can lead to irregular stenosis, thickening, and occlusion of the bronchial wall, manifesting as indirect imaging signs like obstructive pneumonia, emphysema, and atelectasis. Yet, clinicians often misinterpret post-obstructive pneumonia as community-acquired pneumonia, resulting in missed early diagnostic and treatment opportunities for endobronchial tumors. Unlike community-acquired pneumonia, post-obstructive pneumonia stems from bronchial obstruction, causing impaired drainage of distal secretions or atelectasis, sometimes with a secondary infection. The term “postobstructive pneumonia” was coined in the 1970s, replacing “obstructive pneumonitis“ [13]. Both CAP and POP involve lung inflammation, so PCT and CRP levels showed no difference between the two groups in this study. McDonald et al. [14] characterized radiographic opacity from bronchial obstruction by a tumor as post-obstructive pneumonitis. Although research on obstructive pneumonia is limited, evidence suggests many patients initially diagnosed with community-acquired pneumonia actually have underlying lung cancer [15, 16]. Early detection hinges on understanding normal bronchial anatomy, comparing bilateral pulmonary bronchi, and utilizing advanced imaging techniques. Prompt bronchoscopy and biopsy are pivotal for enhancing early diagnosis rates. Certain rare tumors, like lung carcinoids, typically necessitate bronchoscopy for accurate diagnosis due to the limitations of imaging techniques such as CT or PET/CT [17]. Misidentifying intraluminal bronchial tumors and obstructive pneumonia can worsen endobronchial obstruction, increasing the risk of recurrent pulmonary infections, tumor progression, and higher mortality rates. Post-obstructive pneumonia patients often endure longer symptom durations and higher mortality than those with community-acquired pneumonia [18]. While community-acquired pneumonia imaging typically shows consolidation and exudation, obstructive pneumonia features distinct signs like bronchial wall thickening, occlusion, stenosis, obstructive emphysema, bronchial mucus embolism, and intraluminal shadows. Significantly, bronchial occlusion is highly specific for tumor screening, with imaging of bronchial stenosis and/or occlusion aligning closely with bronchoscopic findings for high sensitivity.

Endobronchial tumors include both benign and malignant types, with the latter encompassing squamous cell carcinoma, adenocarcinoma, carcinoid, adenoid cystic carcinoma, and metastatic tumors. Benign varieties consist of hamartomas, neurogenic tumors, and lipomas [19, 20]. Endobronchial resection is often the preferred method for diagnosing and treating these tumors.

Bronchial occlusion and neoplasms were the predominant bronchoscopic findings in patients with post-obstructive pneumonia, representing a significant portion of the cases. In our study, squamous cell carcinoma was the most common pathology, followed by adenocarcinoma. In the POP group, tumor markers associated with squamous carcinoma, such as SCC and CYFRA21-1, were significantly higher than in the CAP group, while the adenocarcinoma-related marker CEA was also significantly higher in the POP group. However, among the 61 patients, only one was diagnosed with small cell lung cancer, which may explain why there was no significant difference in the neuroendocrine tumor marker NSE between the two groups. These results suggest that SCC, CYFRA21-1, and CEA could help indicate the presence of concomitant lung malignancy in pneumonia patients.

However, this study has several limitations. First, it is a single-center study conducted at Affiliated Yueqing Hospital of Wenzhou Medical University, potentially limiting the generalizability of the results to other centers or populations. Furthermore, its retrospective nature means it relies on historical data, which may impact the accuracy of the findings.

In conclusion, There is a possibility of misdiagnosis as community-acquired pneumonia when endobronchial tumors associated with post-obstructive pneumonia. Factors like bronchial occlusion, stenosis, mucus embolism, and intraluminal shadows on CT are significant independent risk factors for endobronchial tumors. Clinicians and radiologists should be vigilant for bronchial abnormalities in pneumonia patients on imaging, particularly when antibiotic treatment is ineffective, and undergo bronchoscopy as early as possible to determine endobronchial tumors.

## Data Availability

The data that support the findings of this study are available from the corresponding author upon reasonable request.
